# Feedback Regulation of Signaling Pathways for Precise Pre-Placodal Ectoderm Formation in Vertebrate Embryos

**DOI:** 10.3390/jdb10030035

**Published:** 2022-08-26

**Authors:** Tatsuo Michiue, Kohei Tsukano

**Affiliations:** Graduate School of Arts and Sciences, The University of Tokyo, 3-8-1, Komaba, Meguro-ku, Tokyo 153-8902, Japan

**Keywords:** morphogen, feedback regulation, signaling pathway, ectoderm, placode

## Abstract

Intracellular signaling pathways are essential to establish embryonic patterning, including embryonic axis formation. Ectodermal patterning is also governed by a series of morphogens. Four ectodermal regions are thought to be controlled by morphogen gradients, but some perturbations are expected to occur during dynamic morphogenetic movement. Therefore, a mechanism to define areas precisely and reproducibly in embryos, including feedback regulation of signaling pathways, is necessary. In this review, we outline ectoderm pattern formation and signaling pathways involved in the establishment of the pre-placodal ectoderm (PPE). We also provide an example of feedback regulation of signaling pathways for robust formation of the PPE, showing the importance of this regulation.

## 1. Introduction

Embryonic patterning is one of the most crucial steps for constructing a complex body shape from a simple egg. The fundamental concept of embryonic fate determination involves localization of signaling molecules inside an egg and differential activation of pathways in each embryonic area, directing localized expression of specific genes. To establish cell fates precisely, strict regulation of signaling strength in each area is essential [1]. There are two types of pattern formation, self-organization and boundary organization [2]. In the “Turing pattern”, a primary example of self-organization, it is possible to create a periodic pattern such as a fish skin pigmentation pattern, simply by employing at least two molecules that differ in diffusion rates and activities [3,4]. This model is very simple; it is impossible to form the precise pattern reproducibly. The second is the so-called “French-flag model”, by which cells generate a pattern due to the strength of a morphogen gradient. This model allows definition of fixed areas more reproducibly than self-organization. In ectoderm patterning, the principle of boundary formation is adopted.

Ectoderm patterning is established after fertilization in vertebrates. The ectoderm consists of four distinct regions, the neural plate (NP), the neural crest (NC), the pre-placodal ectoderm (PPE, also called the pre-placodal region (PPR)), and the epidermis. Patterning is dependent on positional information provided by several types of signaling molecules secreted from mesendodermal tissues. Major signaling types involved in ectodermal patterning include bone morphogenetic protein (BMP), fibroblast growth factor (FGF), retinoic acid (RA), and Wnt. In addition to the ligands themselves, antagonists of each morphogen also contribute to gradient formation in embryos. For example, in *Xenopus* gastrula, several proteins such as chordin, noggin, and follistatin allow formation of BMP gradients. Both FGF and Wnt signaling are important for anterior–posterior neural patterning. Wnt antagonists (dkk, cer, frzb etc.), secreted from anterior mesendoderm, induce anterior neural structure, including the brain [5,6]. PPE formation requires cooperative actions of BMP, FGF, Wnt, and RA signaling to determine the position of the PPE in naïve ectoderm [7].

**Figure 1:**
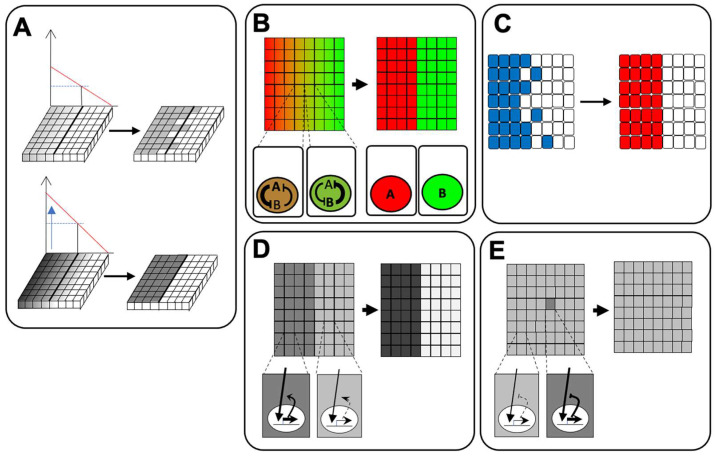
**The strategy for robust pattern formation:** (**A**) steep gradient formation of a morphogen; (**B**) mutual inhibition of transcription factors; (**C**) cell sorting and clear boundary formation of a tissue; (**D**) positive feedback regulation of morphogen gradients; (**E**) negative feedback regulation of morphogen gradients.

The question is whether only the concentration of these molecules enables establishment of the precise ectoderm pattern, because fluctuations of concentration occur, according to various, unexpected environmental factors, resulting in uncertainty in the region of each tissue. To avoid untenable fluctuations, molecular mechanisms must be able to counter such influences. There are several strategies to establish robustness against noise in embryonic patterning (Figure 1).

One of these is establishment of steep gradients (Figure 1A). The larger the difference in morphogen concentration among cells, the more easily each cell is able to detect differences in signal levels [8]. Another strategy is mutual inhibition by two transcription factors (Figure 1B). At an early stage, both genes are expressed in the same cells, whereas expression of one of these genes is decreased, resulting in boundary formation between two regions that each express one of these genes. The third strategy is “cell sorting” (Figure 1C). Gathering cells that receive similar levels of morphogen enables a region to absorb (or average) the noise of patterning, e.g., a salt and pepper cell array around the boundary. The fourth strategy is “local” regulation of signaling, including feedback regulation of intracellular signaling pathways, especially in two regions. Positive feedback regulation makes the two regions more discrete, whereas negative feedback enables them to maintain stable levels of signaling against local turbulence of signal intensity (Figure 1D,E) [2]. Among ectodermal regions, the PPE and the NC are narrow; therefore, a system to precisely form them is more critical than in the NP and the epidermis. In this review, we will focus mainly on PPE formation and will discuss the importance of feedback regulation for local control of appropriate signaling.

## 2. An Outline of PPE Formation

The PPE is a narrow, horseshoe-shaped region induced around the boundary between the neuroectoderm (NE) and the non-neural ectoderm (NNE) during gastrulation [9,10,11]. The NC is also derived from a boundary region and forms craniofacial structures [12,13,14]. The model for dividing the PPE and the NC is discussed later.

**Figure 2:**
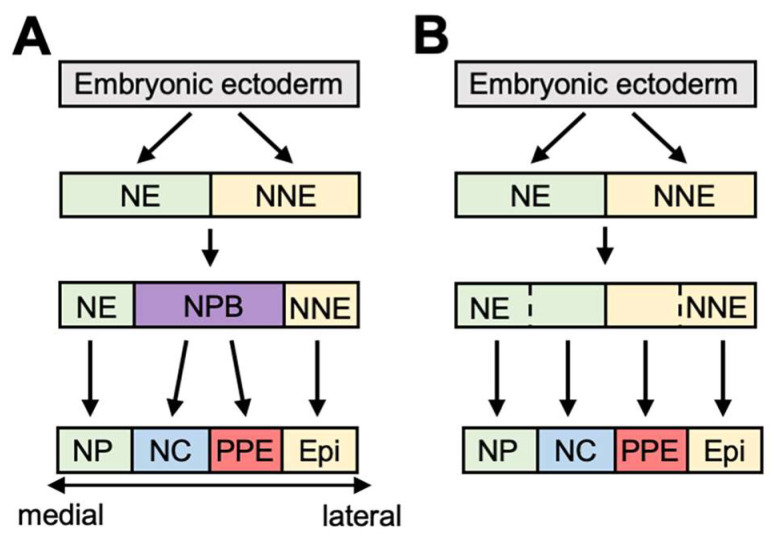
**A model of PPE and NC formation:** (**A**) the neural plate border (NPB) model; Before division of the NC and the PPE, the NPB region is formed between the neuroectoderm and the non-neural ectoderm; (**B**) binary competence model; The NC is derived from the neuroectoderm, whereas the PPE is from the non-neural ectoderm.

PPE cells give rise to cranial sensory organs, including lens, olfactory epithelium, inner ear, some of the cranial ganglia, and the anterior pituitary gland [15,16,17,18,19,20]. In contrast to NC cells, a part of PPE cells remain on the surface of the ectoderm, and after neural tube closure, various patterns of cell migration occur, according to the subtypes of placode [21]. Olfactory epithelium, lens, and otic cells are mainly rearranged to form their final shape, whereas trigeminal and epibranchial cells migrate and aggregate. Many genes are involved in PPE specification and construct a gene network [22]. *Six1*, the homolog of *sine oculis* (*si*) in *Drosophila*, encodes a homeodomain protein and is uniformly expressed in the PPE [23,24]. Eya1 is a cofactor with Six1 and is also expressed in the PPE [25]. These genes are well utilized as pan-placodal markers. The experiment on both upregulation and downregulation has shown that Six1 is required for the gene regulatory network of PPE formation [26,27]. Many other transcription factors including GATA2, Dlx3/5, FoxI1/3 and AP2 are involved with PPE formation (reviewed in [28]). Nonetheless, the molecular mechanism for segregation of the PPE and the NC is controversial in ectoderm patterning, and there are several models to explain PPE/NC formation (Figure 2).

In the “binary competence” model, determination of the neuroectoderm and the non-neural ectoderm occurs during gastrulation, followed by subdivision of the PPE and the epidermis from the non-neural ectoderm, whereas the NC and the NP are derived from the neuroectoderm (Figure 2A). Evidence that supports this model includes the fact that transplantation of NP cells into ventral ectoderm induces Six1, but expression is only seen in the recipient NNE region and not in the donor NP, indicating a difference in competence between the neural and the non-neural ectoderm [6,29]. In addition, Dlx3 plays a role for the formation of differential competence for the PPE [29]. Furthermore, complete inhibition of BMP signaling by dorsomorphin (an antagonist of BMP) at the blastula stage greatly reduced PPE marker expression [30], indicating the importance of at least some BMP signaling at an early stage.

The second model is the “NPB model” (Figure 2B). In this model, the neural plate border (NPB) region is initially induced between the neural and the non-neural ectoderm, followed by subdivision into the NC and the PPE. For NPB formation, several genes are important. *Pax3* and *Zic1* are typical NPB markers. Knockdown of *Zic1* and *Pax3* reduced *Six1* expression, indicating the necessity of both gene functions for PPE formation [31]. The latter study of conserved enhancers revealed that expression of *Pax3* and *Zic1* is regulated by BMP, Wnt, and FGF, and the balance of these signals during the late gastrula stage is essential for *Zic1*/*Pax3* expression [32]. FGF signaling is important for *Pax3* transcription via specific enhancers (called IR2), whereas Wnt signaling positively regulates *zic3* transcription via both E1 and E2 enhancers [32]. *Pax3* expression is positively regulated by itself [33]. Immunostaining with several markers indicates that the PPE and the NC, in addition to the NP, overlap before the neurula stage in chick embryos, supporting this model [34]. Very recently, another model was proposed [35]. The “gradient border model” draws upon both of the previous models. In this model, the neural plate border is induced, but in this area, cells that express NC or PPE genes are distinct, suggesting that NPB already possesses two regions before neurulation.

In the following section, we will discuss intracellular signaling involved in ectoderm patterning. In this patterning, several signaling pathways, including BMP, FGF, Wnt, and RA, participate, but in this review, we focus mainly on BMP and FGF signaling. On the subject of feedback regulation, we will also discuss the implications of RA signaling.

## 3. Control of BMP Signaling in PPE Formation

**Figure 3:**
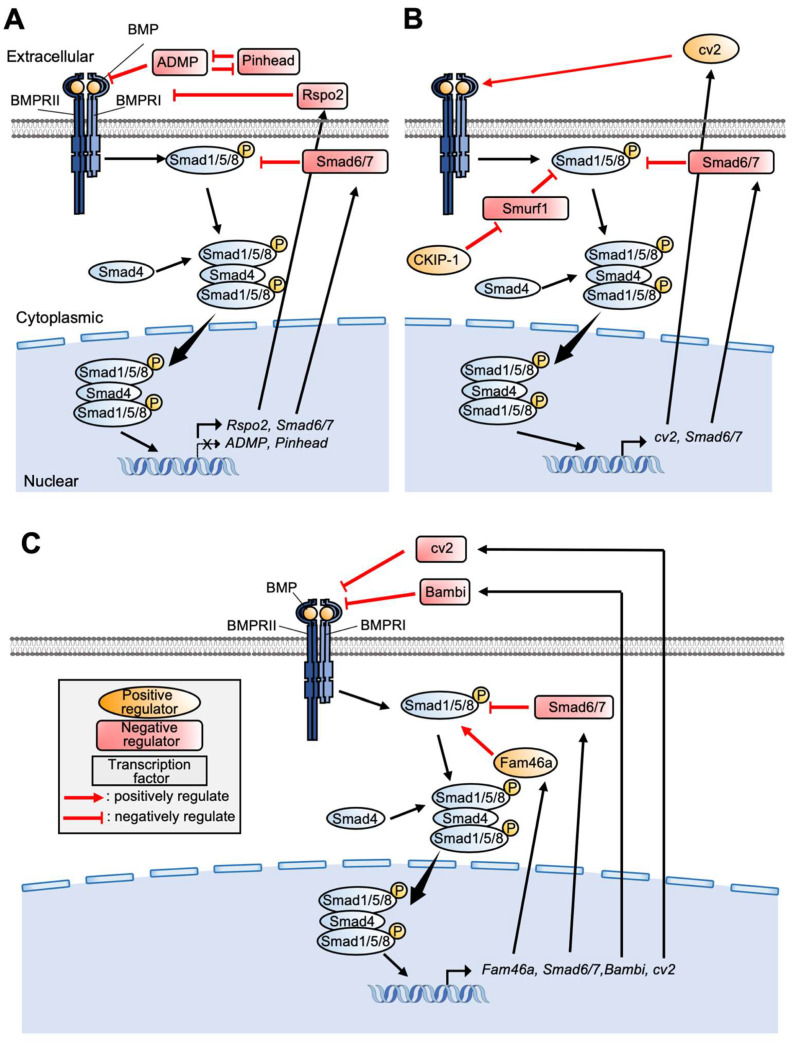
**An outline of the BMP signaling pathway and a list of related factors described in this review:** Factors involved in DV patterning (**A**), NPB formation (**B**), and PPE formation (**C**) are shown.

BMP serves important functions in various biological events, including many kinds of organ development in both vertebrates and invertebrates. Interaction of BMP ligands with BMP receptors promotes phosphorylation of the C-terminal serine residue of Smad1, directing it to bind Smad4, and regulating target gene expression (Figure 3) [5,36].

A morphogen gradient of BMP signaling is essential to establish embryonic patterning, as in dorsoventral axis formation [37]. Similarly, BMP signaling is crucial for ectoderm patterning. BMP4 and 7 are expressed in NNE, next to the PPE [38,39,40,41,42], whereas BMP antagonists are expressed in mesoderm underlying the PPE or in the PPE itself, contributing to differential control of the BMP level [43,44]. Animal cap experiments indicate that NP gene expression decreases as the dose of BMP increases [45]. Despite the fact that determination of the ectodermal region is crucial for precise body plan formation, the molecular mechanism by which BMP morphogen establishes each region is not still fully understood.

For NC formation, various animal models indicate the importance of BMP signaling, although what level of BMP signal is necessary remains controversial. In *Xenopus* embryos, signaling from DLMZ during gastrulation is important, whereas the signal from intermediate mesoderm, as well as adjacent ectoderm is important for maintenance of the NC region, indicating the necessity of stage-dependent inhibition of BMP signaling for NC formation (low BMP level in the early stage, whereas high level in the late stage) [46,47]. On the other hand, positive regulation of BMP signaling is necessary to induce the NC from the neural plate in chick embryos [48,49]. Furthermore, a zebrafish study indicated that intermediate levels of BMP specify a cranial neural crest progenitor [50].

For PPE formation, what function does BMP signaling serve? We need to consider the mechanism along with the binary competence and NPB models described above. According to the NPB model, intermediate levels of BMP signaling during gastrulation and neurulation are necessary for PPE formation, and evidence that supports the NPB model from the point of BMP signaling has been presented. An intermediate level of BMP signaling activity directs PPE induction. In chick embryos, the NPB region shows intermediate intensity of phosphorylated Smad1 protein [51]. A *Xenopus* study using animal cap cells indicated that *Six1* expression is highest with an intermediate dose of noggin or chordin [27,52]. Moreover, *dlx5* and *dlx6* are both expressed in NPB, and the quantitative level of expression was highest in *Xenopus* embryos injected with an intermediate amount of *chordin* (*chd)* mRNA [53]. Another zebrafish study indicated that for PPE formation, a somewhat higher level of BMP signaling is necessary than for the NC [54]. A similar experiment was carried out using zebrafish embryos [55]. In summary, intermediate BMP levels enable induction of NPB/placode gene expression, at least in several experimental systems employing *Xenopus*, zebrafish, and chick embryos.

In the binary state model, it is likely that positive regulation of BMP signaling before gastrulation is important for inducing the PPE, whereas the chick and Xenopus study indicated that attenuation of BMP signaling is necessary at late gastrula/neurula stages to induce the PPE in naïve ectoderm [6,7]. Similarly, using various doses and variable timing of treatments with dorsomorphin, a zebrafish study showed that BMP inhibition at blastula or early gastrula greatly reduced PPE marker expression (*sox3*, *six4* and *pax2*), whereas BMP inhibition at a later stage is important [30]. *Tfap2A/C*, *Fox1i* and *Gata3*, which are necessary to acquire PPE formation competence, are induced by BMP, whereas BMP signaling is not necessary to specify PPE fate after gastrulation [29,30,56]. A chick study also indicated that BMP signaling is required for formation of olfactory and lens placodes [57]. From these studies, it is suggested that during gastrulation BMP promotes PPE formation but subsequently inhibits PPE formation in the non-neural ectoderm.

## 4. Involvement of FGF Signaling for PPE Formation

**Figure 4:**
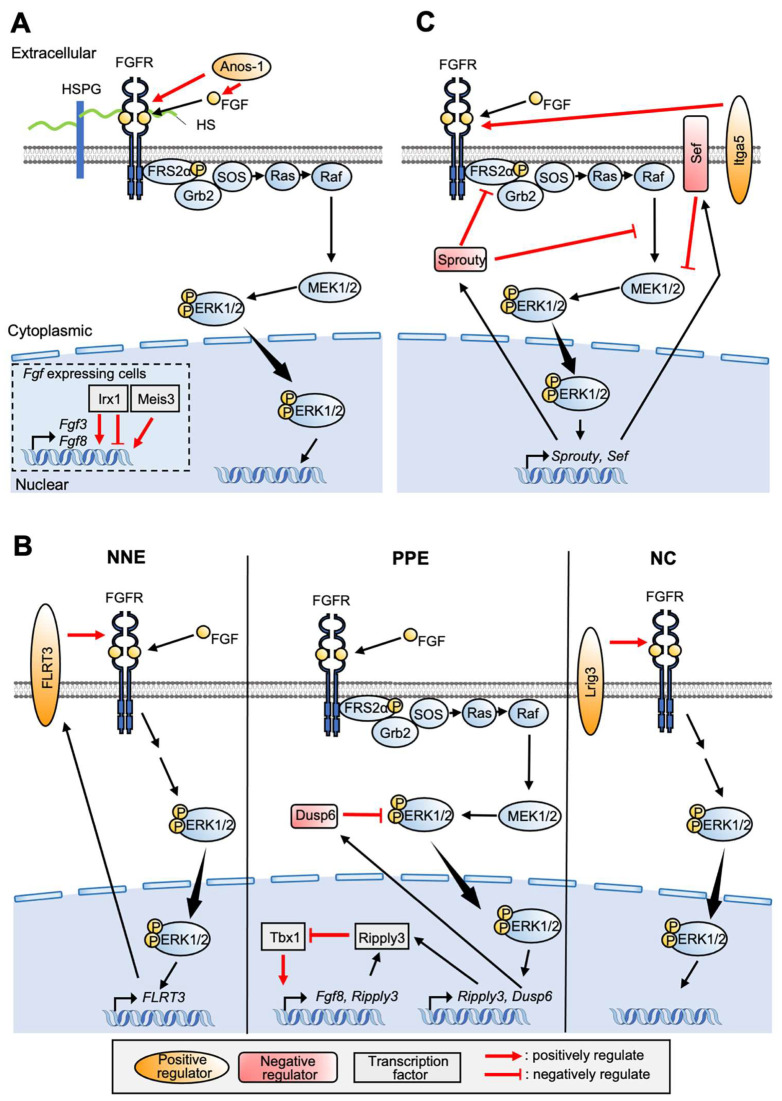
**An outline of the FGF signaling pathway and a list of related factors described in this review:** Factors involved in NPB (**A**), NC/PPE/NNE (**B**), and posterior placode (**C**) formation are shown. Properties of each factor and their actions on targets are shown by color and by arrows. Factors involved in PPE formation are shown in red or orange.

Many studies have reported that relevant genes are involved in NPB/NC formation (Figure 4). Anosmin-1(Anos1), an ECM-associated, glycosylated protein directly interacts with FGF ligands and facilitates FGF8-FGFR1 interaction in chick embryo (Figure 4A) [58,59,60,61]. *Xenopus Anos1* is expressed downstream of *Pax3* and *Zic1* and contributes to formation of both the NC and the PPE [62]. *Meis3* is also expressed downstream of *Zic3* and *Pax3* and positively regulates *Fgf3* and *Fgf8* (Figure 4A) [63]. *Lrig3*, expressed in the NP and the NC, interacts with FGFR1 and modulates FGF signaling in NC induction and specification (Figure 4B) [64]. For establishment of the NC, the balance of ERK and AKT is important [65]. In the NC state of animal cap cells exhibited by *Foxd3* and *Sox9* expression, the pERK level is high and pAKT is low. Thus, NC formation is inhibited by either ERK inhibition or AKT activation [66].

The importance of FGF signaling for PPE formation has also been shown by a series of studies. In *Xenopus* embryos, *Fgf3*, *Fgf4*, and *Fgf8* are expressed in the dorsolateral marginal zone [67]. In chicken blastula, *Fgf8* is distributed in almost all parts of the epiblast, and expression accumulates in the primitive streak at early gastrula stage [68]. Additionally, *Fgf8* is expressed in the anterior neural ridge, adjacent to the PPE [7,69,70]. In *Xenopus* embryos, knockdown of Fgf8 by morpholino anti-sense oligo (MO) decreased *Six1* expression [6,31], and experiments using SU5402 (FGFR inhibitor) also indicated that an FGF signal is required for placode induction [6]. Although FGF signaling is necessary for PPE specification [6,7,31], overactivation of FGF signaling represses a PPE marker gene, *Six1* [27,31]. In addition, slight inhibition of FGF signaling enhances *Six1* expression [52], suggesting that an appropriate level of FGF is required for PPE induction.

*Irx,* which encodes Iroquois homeodomain protein, regulates *Fgf8* expression and is involved in NPB specifier-gene expression, including *Msx1*, *Pax3,* and *Zic1* (Figure 4A) [71]. *Irx1* is upregulated by *Six1* and *Eya1*, whereas *Irx1* promotes *Six1* expression in early PPE formation. Irx1 expression overlaps with that of the NPB gene at first, but expression accumulates only in the PPE region. Interestingly, Irx1 changes to suppress *Six1* expression, suggesting a differential stage-dependent role [71].

For specific placode formation from the pan-placodal domain, FGF signaling is necessary [19,72,73]. Mouse KO experiments also indicate essential roles of Fgf3, Fgf8, and Fgf10 in otic placode formation [73,74]. Integrin-α5 (Itga5) is expressed in the PPE, and its knockdown impaired trigeminal, epibranchial, and otic cells (Figure 4C) [75]. In addition, *dlx3/dlx4* negatively regulates *Fgfr1/2* expression, resulting in malformation of otic placode [44].

## 5. Feedback Regulation of Signaling Pathways for Ectodermal Patterning

As shown above, feedback regulation is a useful way not only to establish discrete areas, but also to maintain levels of intracellular signaling against fluctuations. We will show some examples of feedback regulation in BMP and FGF pathways and their contributions to PPE formation.

### 5.1. BMP Signaling

Several studies have addressed feedback regulation of BMP signaling in the context of embryonic development. In zebrafish embryos, both Pinhead and ADMP encode BMP-like ligands that promote chd degradation, whereas their transcription is repressed by BMP signaling (Figure 3A) [76,77,78]. In *Xenopus* ectoderm formation, R-spondins (RSPOs) antagonize BMP signaling by associating with the BMP receptor, affecting dorsoventral patterning. Biochemical analysis indicates that BMP promotes Rspo2 transcription, whereas RSPO protein antagonizes BMP signaling extracellularly, suggesting feedback loop formation (Figure 3A) [79]. Bambi is induced by BMP4, whereas Bambi represses the ligand–receptor complex, indicating negative loop formation (Figure 3C) [80]. This negative feedback regulation extends the dynamic range of BMP signaling because this system enables responses to more intense BMP signaling, contributing to attenuation of morphogen fluctuation in embryos [81]. Actually, in *Xenopus* embryos, the myf5 expression domain induced by intermediate BMP levels is perturbed by Bambi knockdown.

For PPE/NPB formation, there are not many studies that directly demonstrate the contribution of feedback regulation of BMP signaling. In *Xenopus* embryos, expression of *crossveinless2* (*cv2*), which interacts with both chd and BMP, is seen in high BMP regions, although knockdown of *cv2* with cv2 MO increased *vent1* and *cv2* and decreased *Six3* and *chd*, indicating that cv2 participates in a negative feedback loop of BMP signaling (Figure 3C) [82,83]. On the other hand, the zebrafish study indicates that cv2 forms the positive feedback loop by acting as pro-BMP factor and is required for NC induction by locally enhancing BMP activity and regulating the NPB gene network [84,85]. In the PPE region, dlx3 expression domain is outside the cv2 expression domain in the 5-somite stage of zebrafish embryos. *Dlx3b* enhances *bambi-b* in the PPE, suggesting that discrete expression of these genes specifies both the NC and the PPE region [85]. In chick embryos, *casein kinase interacting protein 1* (*CKIP-1*) and *Smurf1,* which encodes a ubiquitin ligase, are both expressed in NPB and establish an intermediate BMP level with *Smurf1* for NC formation. *Smurf1* attenuates BMP signaling via degradation of Smad1/5/8 but also degrades itself. At the same time, CKIP-1 directly interacts with Smurf1, promoting Smurf1 degradation. In summary, *CKIP-1*/*Smurf1* double-negative attenuation maintains appropriate BMP signal levels in NPB (Figure 3B) [86].

Our analysis indicates the importance of *Fam46a* in PPE formation. Knockdown of *Fam46a* inhibits PPE-specific gene expression, including *Six1*. Fam46a protein directly interacts with the N-half region of Smad1, including the linker domain, and increases the quantitative level of Smad1. The linker region of Smad1 is phosphorylated by GSK3β, followed by ubiquitination and degradation via the proteosome system; thus, it is suggested that Fam46a upregulates BMP signaling via stabilization of Smad1 protein. Moreover, *Fam46a* transcription is promoted by BMP signaling, indicating formation of a positive feedback loop in BMP signaling (Figure 3C). Notably, activation of BMP signaling by Fam46a is not intense because Fam46a contributes to stabilization of Smad1 but not to direct activation via promotion of C-terminal phosphorylation of Smad1 [87].

### 5.2. FGF Signaling

For FGF signaling, feedback controls in either NPB/PPE/specific placode formation have been more widely reported than for BMP signaling. Tbx1 and Ripply3 contribute to regulation of PPE gene expression. In detail, Tbx1 facilitates expression of *Fgf8*, *Six1*, *Eya1,* and *Ripply3*. Additionally, Fgf8 promotes *Ripply3* expression. On the other hand, Ripply3 suppresses expression of *Fgf8* and *Tbx1* and forms a negative feedback loop with Fgf8, Ripply3, and Tbx1 (Figure 4B). This feedback loop contributes to the postero–lateral boundary during formation of the PPE by restricting the expressing region of *fgf* [88]. *Fibronectin-leucine-rich transmembrane protein 3* (*FLRT3*) functions as a positive regulator of Ras-MAPK signaling and also promotes ERK phosphorylation. *FLRT3* transcription is upregulated by FGF signaling, suggesting positive feedback formation (Figure 4B) [89,90]. *Xenopus* FLRT3 is co-expressed with *Fgf8* in the anterior neural ridge. From the fact that overactivation of FGF signaling inhibits PPE formation, FLRT3 may play a role in boundary formation outside the PPE region [90,91]. Recently, we showed that *Dual specificity phosphatase 6* (*Dusp6*, also known as *MKP3*) is important to precisely form the PPE region. Dusp6 is a phosphatase that specifically interacts with dual tyrosine and threonine residues of ERK1/2, attenuating Ras/ERK signaling (Figure 4B) [92,93,94,95]. Our study showed that *Dusp6* is expressed in the PPE at mid-neurula of *Xenopus* embryos in an FGF signal-dependent manner and is necessary for both NPB and PPE formation by modulating FGF signaling. An experiment combining FGF bead transplantation with Dusp6 knockdown demonstrated the importance of negative feedback control for PPE formation. In this study, it was suggested that stable spatial pattern formation against perturbation of FGF ligands is accomplished by suppressing intracellular signaling activity [96].

Furthermore, several genes involved in FGF signaling contribute to specific placode formation. *Sprouty* (*Spry)* functions as an intracellular negative feedback regulator of FGF signaling in several developmental contexts [97,98,99]. *Spry* is expressed in an FGF-dependent manner [100,101], and in mouse embryos, conditional knockout of *Spry1* causes defective craniofacial and cardiac development, indicating the importance of NC formation [102]. *Spry1* and *Spry2* are expressed in posterior PPE and participate in otic placode formation by inhibiting FGF signaling [103,104]. *Spry1* and *Spry2* also contribute to epibranchial placode formation and neuronal differentiation [105]. Malformation of otic placode by *Spry1* and *Spry2* knockdown was rescued by haploinsufficiency of *Fgf8* gene function, suggesting the importance of feedback loop-based fine tuning of FGF signaling (Figure 4C) [105]. *Similar expression of fgf* (*Sef*) regulates Ras-MAPK signaling, as well as other types of signaling [61,106]. In both zebrafish and *Xenopus* embryos, *Sef* is expressed in an FGF signaling-dependent manner, whereas FGF target gene expression is suppressed by *Sef* overexpression. Injection with Sef MO expanded the *Fgf8* expression region in the midbrain–hindbrain boundary (MHB) [107]. In chick embryos, *Sef* is expressed in otic placode [108], suggesting negative feedback loop regulation via Sef, at least in otic placode (Figure 4C).

RA signaling functions cooperatively with FGF signaling. RA nuclear receptor, RARa2, reduced the expression of *Ripply3*, *Tbx1* and *Six1*. As shown above, Ripply3 suppresses FGF signaling, suggesting that RA and FGF signaling form a negative feedback loop via these genes [109]. Pitx2c is induced by RA and promotes transcription of Fgf8, followed by upregulation of Cyp26c1 (an RA metabolizing enzyme) expression adjacent to the PPE. This negative feedback regulation via both FGF and RA signaling suggests a role in PPE specification [110]. In otic vesicle formation, FGF signaling is required for aldh3 (RA synthesizing enzyme) expression, whereas RA treatment itself downregulates fgf8 expression, resulting in feedback loop formation [111].

## 6. Conclusions

In this review, we discussed the role of signaling pathways in PPE formation. In particular, we focused on BMP and FGF signaling and showed examples of their feedback regulation in PPE patterning. Signal adjustment is obviously important not only to form clear boundaries, but also to pattern narrow areas robustly. In particular, feedback adjustment contributes to noise suppression, which reduces signal fluctuation, and contributes to robust acquisition of patterns.

## 7. Future Directions

Further analysis is needed to fully elucidate the mechanisms of formation of the PPE region, the NP, the NC, and the epidermis. In addition, other experimental approaches may be important: one of them is to artificially change the feedback cycle by changing the intron length of a target gene and examining the effect on PPE formation [112]. Furthermore, other principles may need to be considered. One of these is mechanical regulation. Recently, a study using human pluripotent stem cells indicated that NPB fate determination is affected by external forces via changes in BMP signaling [113]. For directional migration of NC cells, a gradient of stiffness in surrounding cells, so-called “durotaxis”, is important [114]. Additionally, our studies indicate that there is a difference in cell tension between neural and epidermal ectoderm [115,116]. From these results, it appears that mechanical forces may contribute to form each ectodermal region and to establish their properties. Another point concerns extracellular control of ligand diffusion. Various molecules, including ECM, membrane protein (receptors, etc.), and other cellular processes, including endocytosis, affect ligand diffusion; thus, these mechanisms are also expected to contribute to ectoderm patterning. Other studies report that ECM protein is involved in NC/PPE formation [70]. In addition, anos1, which associates with FGF ligands, also binds to heparan sulfate (HS) [60,117]. By investigating the contributions of these mechanisms to regulation of intracellular signaling, we will better understand the robust and precise system of embryonic pattern formation.

## Data Availability

Not applicable.
